# Association of Pulmonary Function With Motor Function Trajectories and Disability Progression Among Older Adults: A Long-Term Community-Based Cohort Study

**DOI:** 10.1093/gerona/glac085

**Published:** 2022-05-04

**Authors:** Jingya Wang, Jiao Wang, Xuerui Li, Zhangyu Wang, Xiuying Qi, Abigail Dove, David A Bennett, Weili Xu

**Affiliations:** Department of Epidemiology and Biostatistics, School of Public Health, Tianjin Medical University, Tianjin, China; Tianjin Key Laboratory of Environment, Nutrition and Public Health, Tianjin, China; Department of Epidemiology and Biostatistics, School of Public Health, Tianjin Medical University, Tianjin, China; Tianjin Key Laboratory of Environment, Nutrition and Public Health, Tianjin, China; Center for International Collaborative Research on Environment, Nutrition, and Public Health, Tianjin, China; Department of Epidemiology and Biostatistics, School of Public Health, Tianjin Medical University, Tianjin, China; Tianjin Key Laboratory of Environment, Nutrition and Public Health, Tianjin, China; Center for International Collaborative Research on Environment, Nutrition, and Public Health, Tianjin, China; Department of Epidemiology and Biostatistics, School of Public Health, Tianjin Medical University, Tianjin, China; Tianjin Key Laboratory of Environment, Nutrition and Public Health, Tianjin, China; Center for International Collaborative Research on Environment, Nutrition, and Public Health, Tianjin, China; Department of Epidemiology and Biostatistics, School of Public Health, Tianjin Medical University, Tianjin, China; Tianjin Key Laboratory of Environment, Nutrition and Public Health, Tianjin, China; Center for International Collaborative Research on Environment, Nutrition, and Public Health, Tianjin, China; Aging Research Center, Department of Neurobiology, Care Sciences and Society, Karolinska Institutet, Stockholm, Sweden; Rush Alzheimer’s Disease Center, Rush University Medical Center, Chicago, Illinois, USA; Department of Epidemiology and Biostatistics, School of Public Health, Tianjin Medical University, Tianjin, China; Aging Research Center, Department of Neurobiology, Care Sciences and Society, Karolinska Institutet, Stockholm, Sweden

**Keywords:** Cohort study, Disability, Motor function, Pulmonary function

## Abstract

**Background:**

The association of pulmonary function (PF) with motor function and disability remains unclear. We investigate the association of PF with motor function trajectories and disability progression, and explore the role of social activity, cognitive function, and cardiovascular diseases (CVDs) in this relationship.

**Methods:**

Within the Rush Memory and Aging Project, 1 403 disability-free participants (mean age: 79.28 years) were followed for up to 22 years. PF was measured with a composite score based on peak expiratory flow, forced expiratory volume in 1 second, and forced vital capacity at baseline. Global motor function including dexterity, gait, and hand strength was assessed annually using 10 motor tests. Disability was evaluated according to the basic activities of daily living. Social activity was defined as the frequency of common types of social interaction. Global cognitive function was assessed using a battery of 19 cognitive performance tests. CVDs (including stroke, congestive heart failure, and heart diseases) were ascertained at baseline. Linear mixed-effects models were used.

**Results:**

Compared to high PF, low PF was related to faster decline in global motor function (β = −0.005, 95% confidence interval [CI]: −0.008 to −0.001) and all 3 specific motor abilities (*p* < .05), as well as faster progression of disability (β = 0.012, 95% CI: 0.009 to 0.014). There was a statistically significant interaction between PF and social activity/cognitive function on disability progression (β = 0.005, 95% CI: 0.001 to 0.009, *p* = .010/β = 0.004, 95% CI: 0.001 to 0.009, *p* = .025).

**Conclusion:**

Poor PF accelerates motor function decline and the progression of disability. A high level of social activity and cognitive function appear to decelerate disability progression related to poor PF.

Pulmonary function (PF) is an important parameter for evaluating the health of the respiratory system. Over the lifespan, PF tends to increase until the mid-Twenties and then diminish with age due to modifications in elastic recoil and thorax compliance, and eventually leads to a gradual decrease in the body’s oxygen supply (1). Accumulating evidence has shown that poor PF is predictive of lower quality of life (2), poor cognitive function (3), and an increased risk of dementia (4) and mortality (5). There is also some evidence to support the hypothesis that pulmonary function is associated with physical function in the general population (6,7).

Several studies have reported low levels of individual PF indicators—including peak expiratory flow (PEF), forced expiratory volume in 1 second (FEV1), and forced vital capacity (FVC)―were associated with lower motor function (8–11), but others found no significant association (12–14). A composite indicator of PF combining PEF, FEV1, and FVC can more comprehensively reflect pulmonary respiratory function, as it reflects both lung capacity and respiratory muscle function (15). One previous longitudinal study has shown that poorer lung function, according to a composite indicator, is related to lower gait speed (16). However, the impact of lung function on long-term trajectories of global motor function and specific motor abilities remains unclear.

Disability can occur when motor and/or cognitive function declines to the point that it begins to interfere with an individual’s ability to carry out daily activities (17). Emerging evidence suggests that impaired PF may contribute to disability in older adults (6,18–20), but few studies have addressed this issue using longitudinal data (5,21,22). Cardiovascular diseases (CVDs), lower levels of social activity, and cognitive function are all related to both impaired PF (3,23,24) and disability (25–27) and may play a modulating role in the PF-disability association. To the best of our knowledge, no studies have investigated whether and to what extent poor PF affects the progression of disability among older adults and if this is influenced by social activity, cognitive function, and CVDs.

In the present study, we (a) examined the association between a composite PF indicator and long-term trajectories of global motor function and specific motor abilities; (b) assessed the impact of poor PF on the progression of disability; and (c) explored the joint effects of PF with social activity, cognitive function, or CVDs on disability progression using data from a long-term community-based cohort study.

## Method

### Study Population

The Rush Memory and Aging Project (MAP) is an ongoing community-based longitudinal cohort study of common chronic conditions of aging with an emphasis on declines in cognition and motor function over time (28). Briefly, from 1997 to 2020, a total of 2 192 participants were followed up annually for a maximum of 22 years. After excluding 789 people with prevalent disability (*n* = 260), chronic obstructive pulmonary disease (COPD; *n* = 99), and missing data on baseline PF (*n* = 270) or motor function over follow-up (*n* = 380), 1 403 participants remained for the current study (Supplementary Figure 1).

The study was approved by an Institutional Review Board of Rush University Medical Center and was performed in accordance with the ethical standards laid out in the 1964 Declaration of Helsinki and its later amendments. All participants provided informed consent and a repository consent that allowed their data to be shared.

### Assessment of PF

Using a hand-held spirometer (MicroPlus Spirometer MS03, MicroMedical LTC. Kent, UK), 3 measures of PF were collected from participants at baseline and each follow-up: FVC, FEV1, and PEF (3). Each measure was collected twice, and the values were averaged. These values were further converted into a *z*-score by standardizing the distribution of the measured values according to all participants. The *z*-scores of FVC, FEV1, and PEF were then averaged to yield the composite PF score for each participant, with a higher score indicating better PF. The composite PF score was used as both a continuous and categorical variable (tertiled as low, medium, and high [reference]) in data analyses (15).

### Measurements of Motor Function

Global motor function and specific motor abilities (including dexterity, gait, and hand strength) were assessed at baseline and each follow-up visit using well-established measurement tests (29).

*Dexterity* of the arms was assessed based on the Purdue pegboard test and the finger-tapping test, which respectively measured the number of pegs that could be placed into holes in a pegboard and the rate of index finger tapping on an electronic tapper (Western Psychological Services, Los Angeles, CA) over 10 seconds. Each test was conducted twice for each hand (ie, 4 tests in total), and the measurements were averaged to yield a Purdue Pegboard score and a tapping score, respectively.

*Gait* was evaluated by the amount of time and number of steps required to walk 8 feet and turn 360 degrees. Participants completed this exercise twice, and the measurements of time and number of steps were averaged to obtain four performance scores: walking time, walking steps, turning time, and turning steps.

*Hand strength* was measured in terms of grip strength and pinch strength, measured bilaterally using the Jamar hydraulic hand and pinch dynamometers (Lafayette Instruments, Lafayette, IN). Grip and pinch strength were evaluated twice per hand, and the measurements were averaged to provide an overall grip strength score and pinch strength score. To assess *balance*, we asked participants to stand on 1 leg or their toes for 10 seconds, respectively, and then recorded the maximum time they could hold on to each test (0–10 seconds). We did not form a composite balance measure because the balance tests, unlike the other motor tests, were sometimes not attempted (29).

Raw scores from each test were individually converted to *z*-scores and then averaged to yield a summary global motor score, as detailed in previous studies (29). A higher motor function score indicates better motor function. Composite measures of dexterity (2 tests), gait (4 tests), and hand strength (2 tests) were calculated using the same methodology.

### Definition of Disability

The basic activities of daily living (ADL) as a composite measure of disability consists of 6 items from the Katz Activities of Daily Living Scale, including walking across a small room, bathing, dressing, eating, transferring from bed to chair, and toileting (30). Participants’ ability to complete each of these activities was assessed via self-report at baseline and each follow-up. The level of disability accumulation was quantified by the ADL score (ie, the total number of ADL limitations, ranging from 0 to 6), with higher scores indicating more severe disability. Respondents reporting difficulty with one or more ADL were considered to have a disability.

The instrumental activities of daily living (IADL) is another composite measure of disability that involves a sum of 8 items (telephone use, meal preparation, light housekeeping, heavy housekeeping, handling medications, handling finances, traveling within the community, and shopping) adapted from the Duke Older Americans Resources and Services project (31). Since nearly half of the study participants had an IADL disability at baseline, baseline IADL was only used as a covariate in the sensitivity analysis.

### Assessment of Social Activity, Cognitive Function, and CVDs

Baseline social activity was defined as the frequency with which participants engaged in 6 common types of social interaction over the past year, including (a) going to restaurants, sporting events, or playing games, (b) attending day or overnight trips, (c) engaging in unpaid community/volunteer work, (d) visiting friends or relatives, (e) attending group meetings, and (f) attending church/religious services over the past year. Participants’ level of social activity was dichotomized as low versus high according to the median of the distribution.

Baseline cognitive function was assessed using a battery of 19 cognitive performance tests. Raw scores from the 19 cognitive tests were converted to *z*-scores and then averaged to yield a composite score for global cognitive function (32). The cognitive tests included word list memory, word list recall, word list recognition, immediate and delayed recall of the East Boston Story and Story A from Logical Memory, Boston Naming, category fluency, the National Adult Reading Test, Digits Forward, Digits Backward, Digit Ordering, Judgment of Line Orientation, the Standard Progressive Matrices, the oral version of the Symbol Digit Modalities Test, Number Comparison, and 2 indices from a modified Stroop Neuropsychological Screening Test. The cognitive assessments have been described in further detail previously (3). Participants’ level of cognitive function was dichotomized as low versus high according to the median of the distribution.

CVDs included stroke, congestive heart failure, and heart diseases (including heart attack, coronary thrombosis, coronary occlusion, and myocardial infarction) were ascertained based on neurological or/and medical examination and self-reported medical history.

### Covariates

Information on demographic characteristics, lifestyle factors, anthropometric measures, and medical history was collected at baseline (28). Education was defined as the maximum years of formal schooling. Information on smoking was obtained at baseline through personal interviews, and participants were classified into three categories (nonsmokers, former smokers, and current smokers). Alcohol consumption was defined as the average grams of alcohol consumed per day over the past 12 months (3). Physical activity was operationalized as participants’ total number of hours of physical activity per week based on the National Health Interview Survey, and further dichotomized as low or high according to the median (33).

Height and weight were measured at baseline while participants were wearing light clothing and no shoes. Body mass index (BMI) was calculated as weight (kg) divided by the square of height (m^2^). Blood pressure was recorded as the mean of 2 measurements taken with a mercury sphygmomanometer after participants rested for 5 minutes in a seated position. Hypertension was defined as systolic blood pressure ≥140 mmHg, diastolic blood pressure ≥90 mmHg, or the use of antihypertension drugs.

Diabetes was defined as glycated hemoglobin (HbA1c) ≥6.5%, fasting plasma glucose ≥126 mg/dl, random blood glucose ≥200 mg/dl, self-reported history of diabetes, or the use of diabetes medication. COPD was diagnosed when the FEV1/FVC ratio was ≤0.7 (15).

### Statistical Analysis

Baseline characteristics of the study population by PF levels were compared using chi-square tests for categorical variables and one-way analysis of variance (ANOVA) or Kruskal–Wallis tests for continuous variables.

Linear mixed-effects models were used to estimate the β-coefficients and 95% confidence intervals (CIs) for the associations between PF (as both a continuous and categorical variable) and annual changes in global motor function and specific motor abilities as well as ADL score. The fixed effects included PF, quadratic follow-up time (year^2^), and their interaction based on the distribution of the observed association. The random-effects consisted of random intercept and slope, allowing for the individual differences to be reflected at baseline and over time. Mixed-effect models were first adjusted for age, sex, and education (basic-adjusted) and then further adjusted for smoking, alcohol consumption, physical activity, social activity, cognitive function, BMI, hypertension, diabetes, stroke, congestive heart failure, and heart diseases (multiadjusted). The joint effect of PF and social activity was assessed by creating a dummy variable based on the joint exposures of PF (low vs high) and social activity (low vs high). We examined statistical interaction by incorporating PF, social activity, and their cross-product term in the same model. The same procedure was repeated to examine the joint effect of PF and cognitive function/CVDs on disability progression. The Cox regression model was used to estimate the hazard ratios (HRs) with 95% CIs of the association between PF and disability risk.

In sensitivity analysis, we repeated the analyses after (a) performing multiple imputation for missing values of PF or other covariates at the baseline, (b) excluding 206 participants with incident disability that occurred during the first 3 years of follow-up, (c) additionally adjusting for IADL, and (d) further analysis of the relationship of separate FEV1, FVC, and PEF with motor function and disability. Two-tailed *p* values <.05 were considered to be statistically significant. All statistical analyses were performed using Stata SE 16.0 for Windows (StataCorp, College Station, TX).

## Results

### Baseline Characteristics of the Study Population

At baseline, of the 1 403 disability-free participants (mean age: 79.28 ± 7.44 years, 74.63% female), PF score ranged from −2.22 to 3.42. Compared to those with high PF, participants with low PF were more likely to be older, female, have fewer years of education, lower levels of alcohol consumption, physical activity, and social activity, poor cognitive function and motor functions, and a higher likelihood of hypertension and congestive heart failure. However, there were no significant differences between the 3 PF groups in terms of smoking, BMI, diabetes, stroke, and heart diseases (Table 1).

**Table 1.** Characteristics of the Study Population by Tertiles of Pulmonary Function (PF) at Baseline (*N* = 1 403)

Characteristics	PF^†^			*p* Value
	Low Group (*n* = 462)	Medium Group (*n* = 478)	High Group (*n* = 463)	
Age, years	82.23 ± 6.37	78.79 ± 7.03	76.84 ± 7.84	<.001
Female	431 (93.29)	426 (89.12)	190 (41.04)	<.001
Education, years	14.67 ± 3.04	14.85 ± 2.94	16.11 ± 3.35	<.001
Smoking status				.439
Never smoker	270 (58.57)	295 (61.72)	258 (55.72)	
Former smoker	179 (38.83)	171 (35.77)	194 (41.90)	
Current smoker	12 (2.60)	12 (2.51)	11 (2.38)	
Alcohol consumption, g/d	0.00 (0.00, 5.18)	0.00 (0.00, 4.32)	2.40 (0.00, 10.80)	<.001
Physical activity, h/wk	2.25 (0.67, 4.00)	2.75 (1.00, 5.00)	3.33 (1.75, 6.00)	<.001
Social activity	2.64 ± 0.55	2.75 ± 0.52	2.74 ± 0.56	.007
Cognitive function	0.03 (−0.34, 0.36)	0.17 (−0.20, 0.50)	0.34 (−0.08, 0.65)	<.001
BMI, kg/m^2^	27.50 ± 5.35	27.30 ± 5.12	27.31 ± 5.22	.827
Hypertension	366 (79.22)	342 (71.55)	308 (66.52)	<.001
Diabetes	55 (11.90)	58 (12.13)	72 (15.55)	.412
Stroke	34 (7.76)	36 (8.26)	27 (6.43)	.577
Congestive heart failure	29 (6.28)	16 (3.35)	8 (1.73)	.001
Heart diseases	39 (8.44)	34 (7.13)	42 (9.09)	.535
Global motor function	0.94 ± 0.18	1.05 ± 0.19	1.13 ± 0.20	<.001
Dexterity	0.97 ± 0.15	1.05 ± 0.14	1.08 ± 0.14	<.001
Gait	0.97 ± 0.24	1.05 ± 0.22	1.12 ± 0.25	<.001
Hand strength	0.94 ± 0.30	1.07 ± 0.29	1.14 ± 0.30	<.001

Notes: BMI = body mass index; *SD* = standard deviation. Values are mean ± *SD*, *n* (%), or median (interquartile range). Missing data: alcohol consumption = 1, smoking status = 1, BMI = 12, stroke = 109, congestive heart failure = 1, and heart diseases = 2.

^†^PF category: low group (PF ≤ −0.34), medium group (−0.34 < PF ≤ 0.41), and high group (PF > 0.41).

### Association Between PF and Motor Function

During the follow-up (median: 7 years, interquartile range: 4–9 years), higher PF (as a continuous variable) was dose-dependently related to better global motor function, dexterity, gait, and hand strength over time in multiadjusted mixed-effect models (Table 2).

**Table 2.** β-Coefficients and 95% Confidences Intervals (CIs) for the Association of Pulmonary Function (PF) With Motor Function and Disability Over the Follow-up Period: Results From Linear Mixed-Effects Models

PF	Global Motor Function	Dexterity	Gait	Hand Strength	Disability
	β (95% CI)^†^	β (95% CI)^†^	β (95% CI)^†^	β (95% CI)^†^	β (95% CI)^†^
Baseline					
Continuous PF	0.386* (0.267 to 0.506)	0.297* (0.193 to 0.400)	0.300* (0.151 to 0.449)	0.670* (0.476 to 0.864)	−0.045* (−0.079 to −0.010)
Categories PF					
High	Reference	Reference	Reference	Reference	Reference
Medium	−0.169 (−0.465 to 0.128)	−0.084 (−0.323 to 0.155)	−0.070 (−0.398 to 0.258)	−0.457* (−0.860 to −0.055)	−0.015 (−0.078 to 0.048)
Low	−1.011* (−1.334 to −0.688)	−0.778* (−1.039 to −0.518)	−0.822* (−1.180 to −0.465)	−1.399* (−1.839 to −0.960)	0.078* (0.009 to 0.148)
Longitudinal					
Continuous PF × time2	0.002* (0.000 to 0.003)	0.003* (0.002 to 0.005)	0.002* (0.000 to 0.004)	0.003* (0.001 to 0.005)	−0.005* (−0.006 to −0.004)
Categories PF × time2					
High	Reference	Reference	Reference	Reference	Reference
Medium	0.001 (−0.002 to 0.003)	−0.001 (−0.004 to 0.002)	−0.000 (−0.004 to 0.003)	−0.001 (−0.006 to 0.003)	0.005* (0.003 to 0.006)
Low	−0.005* (−0.008 to −0.001)	−0.006* (−0.010 to −0.003)	−0.005* (−0.009 to −0.001)	−0.006* (−0.011 to −0.001)	0.012* (0.009 to 0.014)

^†^Model adjusted for age, sex, education, smoking, alcohol consumption, physical activity, social activity, cognitive function, body mass index, hypertension, diabetes, stroke, congestive heart failure, and heart diseases.

*p < .05.

**Figure 1.**
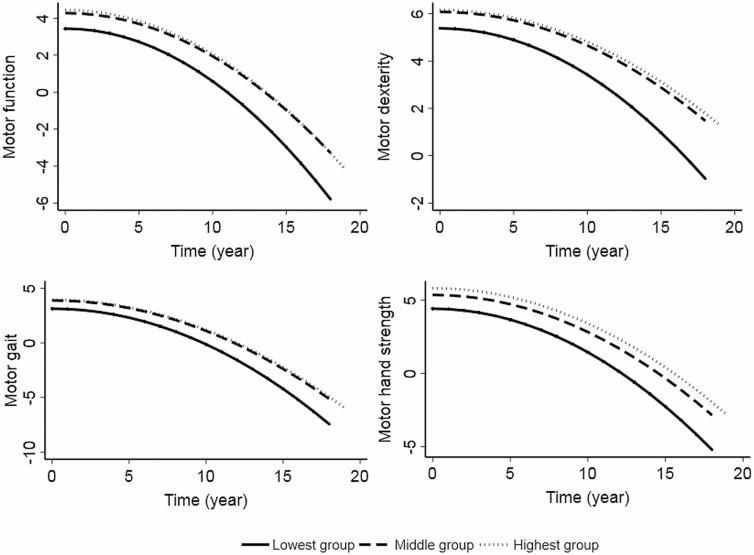
Motor function trajectories over the follow-up period by pulmonary function (PF) tertiled (low, medium, and high). Model adjusted for age, sex, education, smoking, alcohol consumption, physical activity, social activity, cognitive function, body mass index, hypertension, diabetes, stroke, congestive heart failure, and heart diseases.

When PF was used as a categorical variable, compared to high PF, low PF was associated with a faster decline in global motor function, dexterity, gait, and hand strength over the follow-up in multiadjusted mixed-effect models (Table 2 and Figure 1). Results from the basic-adjusted models were similar to those from the multiadjusted models (Supplementary Table 1).

### Association Between PF and Disability Progression

**Figure 2.**
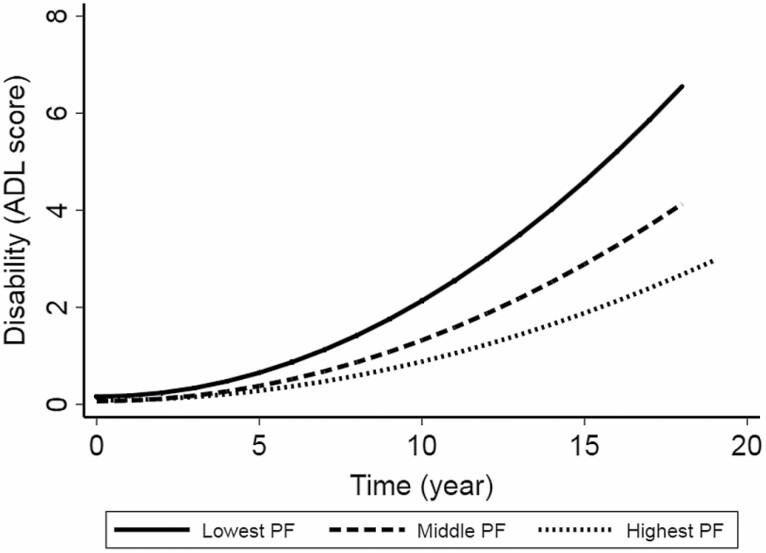
Disability progression over the follow-up period by pulmonary function (PF) tertiled (low, medium, and high). Model adjusted for age, sex, education, smoking, alcohol consumption, physical activity, social activity, cognitive function, body mass index, hypertension, diabetes, stroke, congestive heart failure, and heart diseases.

In the multiadjusted mixed-effect model, higher PF (as a continuous variable) was dose-dependently related to slower disability progression. Compared to high PF, low PF (as a categorical variable) was associated with a faster disability progression after adjusted for age, sex, education, smoking, alcohol consumption, physical activity, social activity, cognitive function, BMI, hypertension, diabetes, stroke, congestive heart failure, and heart diseases (Table 2 and Figure 2). Again, results from the basic-adjusted models were consistent with those from the multiadjusted models (Supplementary Table 1). In the Cox regression model, low PF was associated with a higher risk of disability (HR: 1.38, 95% CI: 1.10 to 1.73; Supplementary Table 2).

### Joint Effect of Poor PF and Social Activity/Cognitive Function/CVDs on Disability Progression

In the joint effect analyses (Table 3), the association between low PF and disability progression was attenuated among people with high social activity/cognitive function and became stronger among people with CVDs. There was a significant multiplicative interaction between low PF and social activity/cognitive function on disability progression (β = 0.005, 95% CI: 0.001 to 0.009*, p =* .010/β = 0.004, 95% CI: 0.001 to 0.009, *p* = .025). No such interaction was present for low PF and CVDs (*p* > 0.05).

**Table 3.** Joint Effects of Poor Pulmonary Function (PF) and Social Activity, Cognitive Function, or Cardiovascular Diseases (CVDs) on Disability Progression

Joint Exposure		Number of Participants	Disability β (95% CI)^†^
Social activity	PF		
High	High	468	Reference
Low	High	473	0.001 (−0.001 to 0.003)
High	Low	210	0.007* (0.004 to 0.010)
Low	Low	252	0.013* (0.010 to 0.016)
*p* Interaction			.009
Cognitive function	PF		
High	High	468	Reference
Low	High	522	0.006* (0.004 to 0.008)
High	Low	183	0.005* (0.002 to 0.008)
Low	Low	279	0.016* (0.014 to 0.019)
*p* Interaction			.025
CVDs	PF		
No	High	802	Reference
Yes	High	139	0.004* (0.001 to 0.007)
No	Low	375	0.009* (0.007 to 0.011)
Yes	Low	87	0.012* (0.007 to 0.016)
*p* Interaction			.624

Note: CI = confidence interval.

^†^Model adjusted for age, sex, education, alcohol consumption, smoking, physical activity, body mass index, hypertension, and diabetes, as well as social activity, cognitive function, and CVDs, if applicable.

*p < .05.

### Sensitivity Analysis

In sensitivity analysis, the results were not much altered when we repeated the analysis after (a) using multiple imputation to account for missing values of PF and some covariates (including alcohol consumption, smoking, BMI, stroke, congestive heart failure, and heart diseases; *n* = 1 604; Supplementary Table 3), (b) excluding 206 participants with incident disability that occurred during the first 3 years of follow-up (*n* = 1 197; Supplementary Table 4), (c) additionally adjusting models for IADL (Supplementary Table 5), and (d) further analysis of the relationship of separate FEV1, FVC, and PEF with motor function and disability (Supplementary Tables 6–8). We additionally examined the joint effect of poor PF and social activity/cognitive function/CVDs on motor function and found no significant interaction (Supplementary Table 9).

## Discussion

In this long-term community-based cohort study of older adults, we found that (a) poor PF was associated with global motor function decline, including dexterity, gait, and hand strength; (b) poor PF accelerated disability progression; and (c) there was a multiplicative interaction between poor PF and social activity/cognitive function on disability progression.

To the best of our knowledge, this is the first study to systematically examine the impact of PF on long-term motor function trajectories and disability progression. Several population-based studies have addressed the association between different indicators of PF and specific motor abilities, but with inconsistent findings (8–13,16). A longitudinal study has shown that low PEF could result in impaired motor function, contributing to subsequent declines in balance and dexterity (8). Another longitudinal study, also based on MAP, indicated that lower lung function, assessed by the composite PF indicator, is related to a 60% higher risk of gait speed limitation (gait speed ≤ 0.55 m/s) (16). Moreover PEF, FEV1, or FVC have been positively associated with hand strength and gait speed in previous cohort studies (9–11), although other studies have failed to detect a significant association between FEV1 or FVC and with hand strength and balance (12–14). The discrepancies could be due to differences in the assessment of PF and motor function. As lung function is complex and multidimensional, we combined FEV1, FVC, and PEF to provide a comprehensive measure of PF. Similarly, the current study used a more stable composite index of global motor function including motor dexterity, gait, and hand strength, better enabling the identification of risk factors for and adverse health consequences of motor decline in old age (34). Using these comprehensive measures, we found that poor PF is independently associated with a faster decline in global motor function and specific motor abilities including dexterity, gait, and hand strength.

We additionally found that poor PF was associated with disability progression, an issue that has been unclear in previous literature. Past studies have examined the relationship between indicators of poor PF (such as FEV1, FVC, and PEF) and disability (5,6,18–22). However, the majority of these studies were cross-sectional (6,18) or only included patients with COPD (19,20). So far, 3 population-based longitudinal studies have explored the association between individual PF indicators and disability, but with inconsistent results (5,21,22). Two of these demonstrated that poor PEF is associated with ADL (5,21), but 1 reported no such association (22).

Social environment has a clear influence on health, and an emerging body of research has explored the connection between social environment and factors such as PF and disability. One 4-year follow-up study demonstrated that social integration (defined as a person’s number of social roles) mitigated age-related loss of lung function (23). Another previous cohort study showed that more frequent social activity was associated with reduced risk of developing disability (26). Furthermore, cognitive function has been associated with both PF and disability. Previous prospective cohort studies have linked higher PF to slower cognitive decline (3), and demonstrated that better cognitive function might reduce the burden of disability (27). However, to the best of our knowledge, the joint effect of PF and social activity/cognitive function on disability progression has not been widely explored. In the current study, we found a significant joint effect between poor PF and social activity/cognitive function, whereby high levels of social activity and cognitive function attenuated the association between low PF and disability progression. Our findings suggest that engagement in social activity or maintaining good cognitive function could be a potential prevention strategy to delay disability in older adults, particularly those with diminished PF.

Similarly, CVD events like stroke and heart disease are relevant to both poor PF and accelerated disability progression (24,25). Stroke has been cross-sectionally related to significantly lower levels of FEV1, FVC, and PEF (24), and a previous longitudinal study demonstrated that stroke may accelerate the rate of disability progression (25). Furthermore, heart disease is one of the most common causes of disability (35), accounting for approximately 13.7% of ADL disabilities according to one study (36). Nevertheless, few studies have investigated the dual role of PF and CVDs on disability progression. In the current study, we failed to find a significant joint effect between poor PF and CVDs. This may be due to the small sample size in joint exposure analysis, and further research is required to clarify the interaction between PF and CVD on disability progression.

There are several mechanisms through which poor PF could lead to motor function decline and faster disability progression. First, the reduced oxygen supply related to poor PF could lead to decreased oxidative metabolism of skeletal muscle, potentially causing inadequate energy supply, hypoxemia, or acidosis (37), ultimately, resulting in muscle endurance decline and motor function decline (38). Second, poor PF has been associated with increased circulating inflammatory markers (such as interleukin-6 and tumor necrosis factor-α) (16), which may accelerate skeletal muscle loss through their catabolic effect on muscle function and suppression of muscle synthesis (25). In addition, systemic inflammation may accelerate the development of atherothrombosis and vascular disease, thereby narrowing the arteries and reducing muscles perfusion (25,39), ultimately inducing motor function decline and disability. Finally, poor PF may lead to an imbalance between energy intake and energy expenditure (40), increasing the likelihood of skeletal muscle dysfunction due to loss of muscle mass and fiber atrophy, which in turn contributes to motor function decline and disability (41,42).

Strengths of this study include the relatively large sample size and long-term follow-up, as well as comprehensive assessments of PF and motor function. Nonetheless, some limitations need to be pointed out. First, the participants in this study were volunteers from the community who were relatively old (mean age: 79.3 years) at study entry, and this may limit the generalizability of the results. However, the characteristics of participants in MAP are generally similar to those in the Rotterdam Study (43), the Honolulu-Asia Aging Study (44), and the Kungsholmen project (45) in terms of demographics and chronic conditions at baseline. Still, caution is required when generalizing our results to younger populations. Second, ADL was evaluated based on self-report, which might be subject to recall bias. However, such bias may be minimized by the use of multiple follow-up data sources. Third, PF and social activity were collected at baseline, and the temporal relationship between PF and social activity is unclear. Additionally, measures of social activity were self-reported and included only 6 common types of activity. Future large population-based cohort studies with comprehensive measures of social activities are warranted to confirm the role of social activities in the lung function–motor function/disability progression. Finally, occupational characteristics such as exposure to toxins and chemicals may influence the association of PF with motor function decline and disability (46), but unfortunately, data on these factors were not available.

In conclusion, our study provides evidence that poor PF accelerates motor function decline (including dexterity, gait, and hand strength) and the progression of disability. High levels of social activity and cognitive function may decelerate disability progression related to poor PF. Our results emphasize the importance of monitoring pulmonary function for early detection and prevention of disability among older adults.

## Supplementary Material

glac085_suppl_Supplementary_Material

## Data Availability

Data can be found and requests for MAP data can be made online at the Rush Alzheimer’s Disease Center Resource Sharing Hub (https://www.radc.rush.edu/).
