# Supplementary material for: Association of Pulmonary Function With Motor Function Trajectories and Disability Progression Among Older Adults: A Long-Term Community-Based Cohort Study
Source: J Gerontol A Biol Sci Med Sci. 2022 May 4;77(12):2524–31. doi: 10.1093/gerona/glac085 (PMC9799204; doi:10.1093/gerona/glac085)
Supplement: glac085_suppl_Supplementary_Material [file glac085_suppl_supplementary_material.pdf]

S. Table 1. β-Coefficients and 95% Confidences Intervals (CIs) for the Association of Pulmonary Function (PF) with Motor Function and Disability over the Follow-up Period: Results from Basic-adjusted Linear Mixed-Effects Models.

S. Table 2. Hazard Ratios (HRs) and 95% Confidences Intervals (CIs) for the Association of Pulmonary Function (PF) with Disability Risk: Results from Cox Regression Models.

S. Table 3. β-Coefficients and 95% Confidences Intervals (CIs) for the Association of Pulmonary Function (PF) with Motor Function and Disability over the Follow-up Period after Multiple Imputation (N=1,604): Results from Linear Mixed-Effects Models.

S. Table 4. β-Coefficients and 95% Confidences Intervals (CIs) for the Association of Pulmonary Function (PF) with Motor Function and Disability over the Follow-up Period after Excluding 206 Participants with Incident Disability that Occurred during the First 3 years of Follow-up (N=1,197): Results from Linear Mixed-Effects Models.

S. Table 5. β-Coefficients and 95% Confidences Intervals (CIs) for the Association of Pulmonary Function (PF) with Motor Function and Disability over the Follow-up Period: Results from Linear Mixed-Effects Models.

S. Table 6. β-Coefficients and 95% Confidences Intervals (CIs) for the Association of Peak Expiratory Flow (PEF) with Motor Function and Disability over the Follow-up Period: Results from Linear Mixed-Effects Models.

S. Table 7. β-Coefficients and 95% Confidences Intervals (CIs) for the Association of Forced Expiratory Volume in 1 second (FEV1) with Motor Function and Disability over the Follow-up Period: Results from Linear Mixed-Effects Models.

S. Table 8. β-Coefficients and 95% Confidences Intervals (CIs) for the Association of Forced Vital Capacity (FVC) with Motor Function and Disability over the Follow-up Period: Results from Linear Mixed-Effects Models.

S. Table 9. Joint Effects of Poor Pulmonary Function (PF) and Social Activity, Cognitive Function or Cardiovascular Diseases (CVDs) on Motor Function (N=925) [a].

S. Figure 1. Flow chart of the Study Population.

S. Table 1. β-Coefficients and 95% Confidences Intervals (CIs) for the Association of Pulmonary Function (PF) with Motor Function and Disability over the Follow-up Period: Results from Basic-adjusted Linear Mixed-Effects Models.

PF	Global Motor Function β (95% CI) [a]	Dexterity β (95% CI) [a]	Gait β (95% CI) [a]	Hand Strength β (95% CI) [a]	Disability β (95% CI) [a]
**Baseline**					
Continuous PF	0.597* (0.477 to 0.717)	0.477* (0.375 to 0.580)	0.538* (0.391 to 0.685)	0.668* (0.483 to 0.852)	-0.059* (-0.090 to -0.027)
Categories PF					
High	Reference	Reference	Reference	Reference	Reference
Medium	-0.376* (-0.726 to -0.027)	-0.282* (-0.564 to -0.000)	-0.286 (-0.646 to 0.075)	-0.534* (-0.990 to -0.077)	0.004 (-0.056 to 0.063)
Low	-1.778* (-2.151 to -1.404)	-1.412* (-1.713 to -1.110)	-1.587* (-1.973 to -1.201)	-1.986* (-2.475 to -1.496)	0.102* (0.037 to 0.166)
**Longitudinal**					
Continuous PF × time[2]	0.002* (0.000 to 0.003)	0.003* (0.002 to 0.004)	0.002* (0.000 to 0.003)	0.003* (0.001 to 0.005)	-0.005* (-0.006 to -0.004)
Categories PF × time[2]					
High	Reference	Reference	Reference	Reference	Reference
Medium	0.001 (-0.002 to 0.003)	-0.000 (-0.003 to 0.003)	-0.001 (-0.004 to 0.003)	-0.002 (-0.006 to 0.002)	0.005* (0.003 to 0.007)
Low	-0.004* (-0.007 to -0.000)	-0.005* (-0.008 to -0.002)	-0.005* (-0.009 to -0.001)	-0.005* (-0.010 to -0.001)	0.011* (0.009 to 0.013)

[a] Model adjusted for age, sex, and education.

* $P < 0.05$.

S. Table 2. Hazard Ratios (HRs) and 95% Confidences Intervals (CIs) for the Association of Pulmonary Function (PF) with Disability Risk: Results from Cox Regression Models.

	**No. of**	**Disability**		
**PF**	**participants**	**No. of cases**	**HR (95%CI)** [a]	**HR (95%CI)** [b]
Continuous	1604	800	0.76 (0.68-0.84)	0.81 (0.73-0.91)
Categorical (tertiles)				
High	530	208	Reference	Reference
Medium	544	273	1.29 (1.04-1.60)	1.22 (0.98-1.51)
Low	530	319	1.58 (1.26-1.98)	1.38 (1.10-1.73)

[a] Model adjusted for age, sex, and education.

[b] Model adjusted for age, sex, education, smoking, alcohol consumption, physical activity, social activity, cognitive function, body mass index, hypertension, diabetes, stroke, congestive heart failure, and heart diseases.

S. Table 3. β-Coefficients and 95% Confidences Intervals (CIs) for the Association of Pulmonary Function (PF) with Motor Function and Disability over the Follow-up Period after Multiple Imputation (N=1,604): Results from Linear Mixed-Effects Models.

**PF**	**Motor Function** β (95% CI) [a]	**Dexterity** β (95% CI) [a]	**Gait** β (95% CI) [a]	**Hand Strength** β (95% CI) [a]	**Disability** β (95% CI) [a]
**Baseline**					
Continuous PF	0.403* (0.291 to 0.515)	0.323* (0.226 to 0.421)	0.355* (0.218 to 0.492)	0.581* (0.410 to 0.752)	-0.032* (-0.062 to -0.001)
Categories PF					
High	Reference	Reference	Reference	Reference	Reference
Medium	-0.237* (-0.446 to -0.027)	-0.082 (-0.323 to 0.158)	-0.175 (-0.492 to 0.142)	-0.400* (-0.765 to -0.035)	-0.021 (-0.078 to 0.036)
Low	-0.621* (-0.851 to -0.392)	-0.914* (-1.175 to -0.653)	-1.147* (-1.491 to -0.803)	-1.432* (-1.829 to -1.036)	0.045 (-0.017 to 0.107)
**Longitudinal**					
Continuous PF×time^2	0.000 (-0.001 to 0.001)	0.001* (0.000 to 0.003)	0.001 (-0.000 to 0.002)	0.001 (-0.001 to 0.002)	-0.003* (-0.004 to -0.002)
Categories PF×time^2					
High	Reference	Reference	Reference	Reference	Reference
Medium	-0.021* (-0.022 to -0.020)	-0.015* (-0.016 to -0.013)	-0.027* (-0.029 to -0.025)	-0.024* (-0.026 to -0.021)	0.005* (0.003 to 0.007)
Low	-0.019* (-0.020 to -0.018)	-0.014* (-0.016 to -0.012)	-0.028* (-0.030 to -0.025)	-0.021* (-0.024 to -0.018)	0.006* (0.004 to 0.008)

[a] Model adjusted for age, sex, education, smoking, alcohol consumption, physical activity, social activity, cognitive function, body mass index, hypertension, diabetes, stroke, congestive heart failure, and heart diseases.

* $P < 0.05$.

S. Table 4. β-Coefficients and 95% Confidences Intervals (CIs) for the Association of Pulmonary Function (PF) with Motor Function and Disability over the Follow-up Period after Excluding 206 Participants with Incident Disability that Occurred during the First 3 years of Follow-up (N=1,197): Results from Linear Mixed-Effects Models.

PF	Motor Function β (95% CI) [a]	Dexterity β (95% CI) [a]	Gait β (95% CI) [a]	Hand Strength β (95% CI) [a]	Disability β (95% CI) [a]
**Baseline**					
Continuous PF	0.329* (0.181 to 0.477)	0.293* (0.164 to 0.421)	0.258* (0.075 to 0.441)	0.575* (0.338 to 0.812)	-0.035 (-0.093 to 0.023)
Categories PF					
High	Reference	Reference	Reference	Reference	Reference
Medium	-0.170 (-0.524 to 0.183)	-0.114 (-0.402 to 0.173)	-0.018 (-0.408 to 0.373)	-0.335 (-0.814 to 0.143)	-0.008 (-0.113 to 0.097)
Low	-0.965* (-1.353 to -0.577)	-0.819* (-1.134 to -0.505)	-0.734* (-1.162 to -0.306)	-1.214* (-1.739 to -0.689)	0.084 (-0.032 to 0.201)
**Longitudinal**					
Continuous PF×time[2]	0.001 (-0.000 to 0.003)	0.003* (0.001 to 0.004)	0.002* (0.000 to 0.004)	0.002* (0.000 to 0.004)	-0.005* (-0.0046 to -0.004)
Categories PF×time[2]					
High	Reference	Reference	Reference	Reference	Reference
Medium	0.000 (-0.003 to 0.003)	-0.001 (-0.004 to 0.002)	-0.001 (-0.004 to 0.003)	-0.002 (-0.006 to 0.003)	0.005* (0.003 to 0.007)
Low	-0.004* (-0.007 to -0.000)	-0.005* (-0.008 to -0.002)	-0.005* (-0.009 to -0.000)	-0.005* (-0.010 to 0.001)	0.012* (0.009 to 0.014)

[a] Model adjusted for age, sex, education, smoking, alcohol consumption, physical activity, social activity, cognitive function, body mass index, hypertension, diabetes, stroke, congestive heart failure, and heart diseases.

* $P < 0.05$.

S. Table 5. β-Coefficients and 95% Confidences Intervals (CIs) for the Association of Pulmonary Function (PF) with Motor Function and Disability over the Follow-up Period: Results from Linear Mixed-Effects Models.

PF	Global Motor Function β (95% CI) [a]	Dexterity β (95% CI) [a]	Gait β (95% CI) [a]	Hand Strength β (95% CI) [a]	Disability β (95% CI) [a]
**Baseline**					
Continuous PF	0.430* (0.312 to 0.548)	0.359* (0.254 to 0.463)	0.335* (0.189 to 0.482)	0.660* (0.470 to 0.849)	-0.033* (-0.066 to -0.000)
Categories PF					
High	Reference	Reference	Reference	Reference	Reference
Medium	-0.243 (-0.536 to 0.050)	-0.164 (-0.404 to 0.076)	-0.142 (-0.466 to 0.182)	-0.494* (-0.889 to -0.098)	-0.018 (-0.080 to 0.044)
Low	-1.030* (-1.349 to -0.710)	-0.839* (-1.101 to -0.577)	-0.830* (-1.183 to -0.477)	-1.335* (-1.767 to -0.903)	0.050 (-0.017 to 0.118)
**Longitudinal**					
Continuous PF × $time^2$	0.002* (0.000 to 0.003)	0.003* (0.002 to 0.005)	0.002* (0.000 to 0.004)	0.003* (0.001 to 0.005)	-0.005* (-0.006 to -0.004)
Categories PF × $time^2$					
High	Reference	Reference	Reference	Reference	Reference
Medium	0.001 (-0.002 to 0.003)	-0.001 (-0.004 to 0.002)	-0.000 (-0.004 to 0.003)	-0.001 (-0.006 to 0.003)	0.005* (0.003 to 0.006)
Low	-0.005* (-0.008 to -0.001)	-0.006* (-0.009 to -0.003)	-0.005* (-0.010 to -0.001)	-0.006* (-0.011 to -0.001)	0.012* (0.009 to 0.014)

[a] Model adjusted for age, sex, education, smoking, alcohol consumption, physical activity, social activity, cognitive function, body mass index, hypertension, diabetes, stroke, congestive heart failure, heart diseases, and instrumental activities of daily living.

* $P < 0.05$.

S. Table 6. β-Coefficients and 95% Confidences Intervals (CIs) for the Association of Peak Expiratory Flow (PEF) with Motor Function and Disability over the Follow-up Period: Results from Linear Mixed-Effects Models.

PEF	Global Motor Function β (95% CI) [a]	Dexterity β (95% CI) [a]	Gait β (95% CI) [a]	Hand Strength β (95% CI) [a]	Disability β (95% CI) [a]
**Baseline**					
Continuous PEF	0.489* (0.389 to 0.588)	0.388* (0.300 to 0.476)	0.369* (0.246 to 0.491)	0.632* (0.473 to 0.790)	-0.050* (-0.078 to -0.023)
Categories PEF					
High	Reference	Reference	Reference	Reference	Reference
Medium	-0.166 (-0.456 to 0.124)	-0.100 (-0.336 to 0.137)	-0.183 (-0.499 to 0.134)	-0.645* (-1.031 to -0.259)	0.010 (-0.050 to 0.070)
Low	-1.013* (-1.317 to -0.709)	-0.792* (-1.040 to -0.543)	-0.739* (-1.072 to -0.406)	-1.309* (-1.715 to -0.903)	0.100* (0.037 to 0.164)
**Longitudinal**					
Continuous PEF × time[2]	0.001 (-0.000 to 0.002)	0.003* (0.001 to 0.004)	0.002* (0.000 to 0.003)	0.003* (0.001 to 0.004)	-0.005* (-0.006 to -0.004)
Categories PEF × time[2]					
High	Reference	Reference	Reference	Reference	Reference
Medium	-0.005* (-0.008 to -0.002)	-0.004* (-0.007 to -0.001)	-0.004* (-0.008 to -0.001)	-0.003 (-0.008 to 0.001)	0.006* (0.004 to 0.008)
Low	-0.002 (-0.005 to 0.001)	-0.006* (-0.009 to -0.003)	-0.004 (-0.000 to 0.000)	-0.006* (-0.010 to -0.001)	0.011* (0.009 to 0.013)

[a] Model adjusted for age, sex, education, smoking, alcohol consumption, physical activity, social activity, cognitive function, body mass index, hypertension, diabetes, stroke, congestive heart failure, and heart diseases.

* $P < 0.05$.

S. Table 7. β-Coefficients and 95% Confidences Intervals (CIs) for the Association of Forced Expiratory Volume in 1 second (FEV1) with Motor Function and Disability over the Follow-up Period: Results from Linear Mixed-Effects Models.

**FEV1**	**Global Motor Function** β (95% CI) [a]	**Dexterity** β (95% CI) [a]	**Gait** β (95% CI) [a]	**Hand Strength** β (95% CI) [a]	**Disability** β (95% CI) [a]
**Baseline**					
Continuous FEV1	0.331* (0.223 to 0.439)	0.301* (0.207 to 0.395)	0.255* (0.124 to 0.386)	0.533* (0.363 to 0.703)	-0.037* (-0.066 to -0.007)
Categories FEV1					
High	Reference	Reference	Reference	Reference	Reference
Medium	-0.207 (-0.508 to 0.094)	-0.158 (-0.402 to 0.086)	-0.047 (-0.374 to 0.280)	-0.379 (-0.778 to 0.021)	-0.010 (-0.073 to 0.052)
Low	-1.035* (-1.355 to -0.715)	-0.929* (-1.188 to -0.669)	-0.827* (-1.175 to -0.480)	-1.390* (-1.815 to -0.964)	0.075* (0.009 to 0.142)
**Longitudinal**					
Continuous FEV1 × $time^2$	0.002* (0.000 to 0.003)	0.003* (0.002 to 0.004)	0.002* (0.000 to 0.003)	0.002* (0.000 to 0.004)	-0.005* (-0.005 to -0.004)
Categories FEV1 × $time^2$					
High	Reference	Reference	Reference	Reference	Reference
Medium	0.000 (-0.003 to 0.003)	-0.002 (-0.005 to 0.001)	0.000 (-0.004 to 0.004)	-0.001 (-0.005 to 0.004)	0.005* (0.003 to 0.007)
Low	-0.005* (-0.009 to -0.002)	-0.007* (-0.010 to -0.003)	-0.005* (-0.009 to -0.001)	-0.006* (-0.010 to -0.001)	0.011* (0.008 to 0.013)

[a] Model adjusted for age, sex, education, smoking, alcohol consumption, physical activity, social activity, cognitive function, body mass index, hypertension, diabetes, stroke, congestive heart failure, and heart diseases.

* $P < 0.05$.

S. Table 8. β-Coefficients and 95% Confidences Intervals (CIs) for the Association of Forced Vital Capacity (FVC) with Motor Function and Disability over the Follow-up Period: Results from Linear Mixed-Effects Models.

FVC	**Global Motor Function** β (95% CI) [a]	**Dexterity** β (95% CI) [a]	**Gait** β (95% CI) [a]	**Hand Strength** β (95% CI) [a]	**Disability** β (95% CI) [a]
**Baseline**					
Continuous FVC	0.286* (0.179 to 0.393)	0.251* (0.157 to 0.344)	0.269* (0.139 to 0.398)	0.479* (0.311 to 0.647)	-0.034* (-0.063 to -0.005)
Categories FVC					
High	Reference	Reference	Reference	Reference	Reference
Medium	-0.277 (-0.577 to 0.024)	-0.211 (-0.456 to 0.034)	-0.183 (-0.509 to 0.143)	-0.456* (-0.853 to -0.059)	0.034 (-0.028 to 0.096)
Low	-0.870* (-1.187 to -0.552)	-0.747* (-1.005 to -0.489)	-0.729* (-1.073 to -0.385)	-1.310* (-1.730 to -0.891)	0.081* (0.015 to 0.146)
**Longitudinal**					
Continuous FVC × time[2]	0.001* (0.000 to 0.003)	0.002* (0.001 to 0.004)	0.001* (-0.000 to 0.003)	0.002* (0.000 to 0.004)	-0.004* (-0.005 to -0.003)
Categories FVC × time[2]					
High	Reference	Reference	Reference	Reference	Reference
Medium	0.002 (-0.001 to 0.005)	0.001 (-0.002 to 0.004)	0.001 (-0.002 to 0.005)	0.001 (-0.003 to 0.006)	0.004* (0.002 to 0.006)
Low	-0.004* (-0.008 to -0.001)	-0.005* (-0.008 to -0.002)	-0.005* (-0.009 to -0.001)	-0.003 (-0.008 to 0.002)	0.010* (0.008 to 0.012)

[a] Model adjusted for age, sex, education, smoking, alcohol consumption, physical activity, social activity, cognitive function, body mass index, hypertension, diabetes, stroke, congestive heart failure, and heart diseases.

* $P < 0.05$.

S. Table 9. Joint Effects of Poor Pulmonary Function (PF) and Social Activity, Cognitive Function or Cardiovascular Diseases (CVDs) on Motor Function (N=925) [a].

Joint Exposure		No. of subjects	Global Motor Function β (95% CI) [b]	Dexterity β (95% CI) [b]	Gait β (95% CI) [b]	Hand Strength β (95% CI) [b]
**Social activity**	**PF**					
High	High	231	Reference	Reference	Reference	Reference
Low	High	232	0.000 (-0.004 to 0.004)	0.004 (0.000 to 0.008)	-0.000 (-0.005 to 0.005)	-0.004 (-0.010 to 0.003)
High	Low	210	-0.003 (-0.007 to 0.002)	-0.004 (-0.008 to 0.000)	-0.002 (-0.007 to 0.004)	-0.004 (-0.010 to 0.002)
Low	Low	252	-0.006* (-0.012 to -0.001)	-0.005* (-0.010 to -0.000)	-0.011* (-0.017 to -0.004)	-0.010* (-0.017 to -0.003)
P-interaction			0.286	0.059	0.106	0.675
**Cognitive function**	**PF**					
High	High	283	Reference	Reference	Reference	Reference
Low	High	180	-0.005* (-0.008 to -0.002)	-0.005* (-0.008 to -0.002)	-0.006* (-0.010 to -0.003)	-0.004 (-0.009 to 0.000)
High	Low	183	-0.004 (-0.008 to 0.000)	-0.006* (-0.011 to -0.003)	-0.005* (-0.010 to -0.004)	-0.001 (-0.007 to 0.004)
Low	Low	279	-0.008* (-0.012 to -0.003)	-0.008* (-0.012 to -0.004)	-0.008* (-0.014 to -0.003)	-0.009* (-0.015 to -0.004)
P-interaction			0.652	0.193	0.349	0.360
**CVDs**	**PF**					
No	High	399	Reference	Reference	Reference	Reference
Yes	High	64	-0.005 (-0.011 to 0.002)	-0.006* (-0.012 to -0.000)	-0.009* (-0.017 to -0.001)	-0.002 (-0.011 to 0.007)
No	Low	375	-0.004* (-0.008 to -0.001)	-0.007* (-0.011 to -0.003)	-0.005* (-0.010 to -0.001)	-0.005 (-0.011 to 0.000)

Yes	Low	87	-0.010* (-0.017 to -0.002)	-0.007 (-0.013 to 0.000)	-0.013* (-0.022 to -0.003)	-0.009 (-0.019 to 0.001)
P-interaction			0.918	0.116	0.863	0.623

Note: CI, confidence interval.

[a] In the joint effect analysis, we excluded participants with the medium PF, as only the low PF was related to motor function decline.

[b] Model adjusted for age, sex, education, smoking, alcohol consumption, physical activity, cognitive function, body mass index, hypertension, and diabetes, as well as social activity and CVDs, if applicable.

* $P < 0.05$.

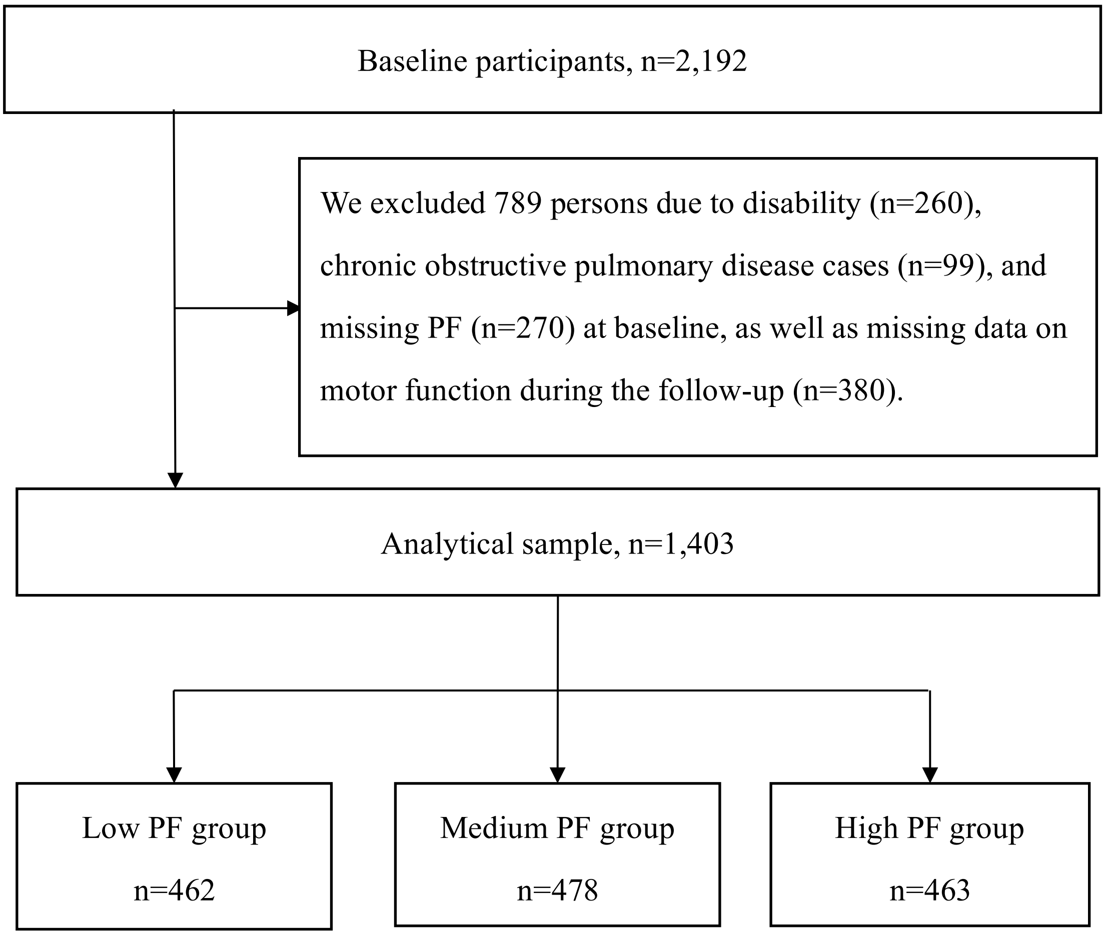

S. Figure 1. Flow chart of the Study Population.

Note: PF, pulmonary function.
